# Osteopontin Levels in Maternal Serum, Cord Blood, and Breast Milk According to Gestational Diabetes Mellitus: A Case-Control Study

**DOI:** 10.3390/nu16244334

**Published:** 2024-12-16

**Authors:** Merve Küçükoğlu Keser, Dilek Şahin, Sıddika Songül Yalçın

**Affiliations:** 1 Department of Pediatrics, Ankara Bilkent City Hospital, Ankara 06800, Turkey; 2 Department of Social Pediatrics, Institute of Child Health, Hacettepe University, Ankara 06100, Turkey; 3 Department of Perinatology, University of Health Sciences, Ankara Bilkent City Hospital, Ankara 06800, Turkey; dilekuygur@gmail.com

**Keywords:** betatrophin, gestational diabetes mellitus, osteopontin

## Abstract

Background/Objectives: The prevalence of gestational diabetes mellitus (GDM) is increasing, and GDM poses health risks for both mothers and newborns. This study investigated the association between GDM and two biomarkers, osteopontin (OPN) and betatrophin (ANGPTL8). Methods: This study involved face-to-face interviews with 165 participants—81 in the GDM group and 84 in the control group—to gather maternal-newborn data. Maternal serum OPN levels, along with cord serum OPN and ANGPTL8 levels, were measured at birth. OPN levels in breast milk were also measured between postnatal days 10–15. Statistical analysis included Student’s t-test for comparing biomarker levels, chi-square tests for GDM incidence across OPN quartile levels, multiple logistic regression for assessing GDM frequency by OPN quartile levels, and the Pearson correlation coefficient to explore relationships between biomarker levels and maternal-newborn characteristics. Results: No significant differences in cord OPN or ANGPTL8 levels were detected between the groups. However, the OPN levels in maternal serum and breast milk were greater in the GDM group than in the control group. We found an association between increasing maternal serum and breast milk OPN levels in quartile groups and the risk of GDM. Additionally, there was a moderate correlation between cord OPN and both maternal serum OPN (r = 0.45) and breast milk OPN (r = 0.43). Conclusions: The differences in OPN levels between the GDM and control groups suggest that OPN may reflect metabolic changes associated with GDM, possibly as a consequence of the condition itself or its treatment. Further research is necessary to validate these findings and uncover the underlying mechanisms involved.

## 1. Introduction

Gestational diabetes mellitus (GDM), which is characterized by varying degrees of carbohydrate intolerance during pregnancy, poses significant risks to maternal and fetal health [1]. It is linked to serious perinatal outcomes such as macrosomia, birth trauma, hypoglycemia, and increased rates of cesarean section (C/S), along with long-term adverse effects such as type 2 diabetes mellitus (T2DM), obesity, and metabolic issues for both mothers and infants [2,3,4]. In 2021, approximately 21.1 million live births, accounting for 16.7% of pregnancies among women aged 20–49 globally, were affected by elevated blood glucose levels during pregnancy [5].

The identified risk factors include advanced maternal age, a high prepregnancy body mass index (BMI), and a family history of diabetes [6,7]. Mechanistically, GDM arises from impaired pancreatic cell function, particularly in the second and third trimesters, often culminating in insulin resistance mediated by proinflammatory cytokines [8]. Research endeavors to elucidate bioactive components contributing to GDM predictability, pathogenesis, and prognosis. Osteopontin (OPN), recognized as a proinflammatory cytokine, and betatrophin (ANGPTL8), a newly identified hormone, have emerged as potential candidates [9,10].

OPN, a acidic phosphoprotein, is synthesized in various tissues and is secreted into body fluids such as blood, urine, and breast milk. It plays a multifaceted role in inflammation, biomineralization, cell viability, and wound healing, with associations noted in cardiovascular diseases, various cancers, diabetes and obesity [11,12]. OPN exists in three isoforms (OPNa, OPNb and OPNc). In 2015, it was shown that there was a significant relationship between serum OPNc and diabetes and/or obesity in patients who had undergone pancreatic surgery [13]. OPN was observed to be expressed in pancreatic islets in nonobese diabetic mice and streptozotocin-induced diabetic (SID) rats [14,15]. Serum OPN levels have been shown to increase in patients with type 1 diabetes mellitus (T1DM), and higher OPN concentrations are associated with a dysmetabolic profile [16]. A study in pediatric patients revealed that high OPN levels are independently associated with T1DM, suggesting a potential role in predicting microvascular diabetic complications [17]. Animal models exposed to high-fat diets have shown increased expressions of OPN in adipose tissue macrophages, which is correlated with elevated plasma OPN levels and subsequent inflammation leading to obesity and insulin resistance [18]. Similarly, a study conducted in overweight/obese individuals demonstrated increased plasma OPN levels and mRNA expression in omental adipose tissue, with further elevation observed in individuals with obesity-associated diabetes [19]. Nevertheless, the relationship between OPN and the development and progression of GDM is not fully understood. Although experimental evidence suggests its involvement in GDM-associated inflammation, with potential therapeutic implications demonstrated in rodent models [20], human studies have reported inconsistent findings for GDM and OPN. In one of the studies, no significant association was found between OPN levels and insulin resistance in GDM patients, and OPN levels were linked to inflammatory markers and liver enzymes [21]. Similarly, two other investigations have not shown a significant relationship between OPN and GDM [22,23]. OPN is expressed in various tissues and body fluids, including blood, urine, and breast milk [16,24]. OPN is regarded as important in the growth of infants [25].

ANGPTL8, which is primarily expressed in the liver and secreted under conditions of insulin resistance, has garnered increasing attention for its involvement in glucose and lipid metabolism [26,27]. The overexpression of ANGPTL8 in SID mice resulted in an increase in β-cell numbers and insulin production and a decrease in glucose levels [10]. However, studies in pregnant women have yielded conflicting results regarding the association between ANGPTL8 levels and GDM, with some suggesting a positive association [28,29,30,31] and one reporting no significant relationship [32]. Additionally, research on the relationships between ANGPTL8 levels and diabetes and its complications remains scarce [31,33,34,35]. Furthermore, it has been shown that a high concentration of ANGPTL8 during the diagnosis of GDM is associated with a greater risk of T2DM in the postpartum period [36]. Moreover, studies indicate that ANGPTL8 levels are elevated in both maternal and cord blood of GDM patients, suggesting a potential role in reflecting intrauterine growth and fetal development [31,33,37]. On the other hand, ANGPTL8 is also secreted by the fetus itself [38]. Taken together, these findings suggest that circulating ANGPTL8 levels could serve as potential biomarkers for GDM, with implications for its diagnosis and prognosis.

We hypothesized that OPN and ANGPTL8 levels will change in GDM-diagnosed mothers compared to those with a normal pregnancy course. Given the inconsistent findings in the literature, our study also aimed to compare OPN levels in three biomatrices [serum, cord blood, and postpartum breast milk (Day 10–15)] with ANGPTL8 levels in cord blood between mothers diagnosed with GDM and pregnant mothers with a normal course. Additionally, we plan to evaluate the relationships between these biomarkers and factors such as maternal anthropometric measurements, gestational weight gain, maternal body fat percentage, and infant birth weight. We anticipate that the results of this study will provide new insights into the etiology and monitoring of GDM.

## 2. Materials and Methods

### 2.1. Study Period

The population of this case-control study included pregnant women who delivered at the Perinatology and Neonatology Clinic of Ankara Bilkent City Hospital, Health Sciences University, between 1 August 2021 and 1 August 2022.

### 2.2. Sample Size

The sample size was calculated so that to detect a situation with an effect size of 0.5 (medium) with a two-way hypothesis with 90% power, 0.05 error, and a 1/1 case/control ratio, a total of 172 mothers, 86 mothers in each group, was necessary (G*Power Version, 3.1.9.4, Gachenbach, Germany).

### 2.3. Study Groups

GDM Group: Pregnant women who presented to the perinatology outpatient clinic between the 24th and 28th weeks of pregnancy and who received a first-time diagnosis of GDM according to diabetes guidelines by a perinatology specialist were eligible for inclusion in this study. Pregnant women undergoing either dietary or insulin treatment were included without discrimination. Infants born at 37 weeks of gestation or later were eligible for inclusion, irrespective of being small for gestational age (SGA), appropriate for gestational age (AGA), or large for gestational age (LGA). Pregnant women with a prior diagnosis of diabetes mellitus, those with twin pregnancies, and those who did not provide informed consent were excluded from the GDM group.

Control Group: Each pregnant woman who was diagnosed with GDM and included in the case group was matched with a control group consisting of a woman who delivered at the clinic on the same day, had no previous diagnosis of GDM or diabetes mellitus (DM), and had a singleton birth at 37 weeks of gestation or later.

**Figure 1:**
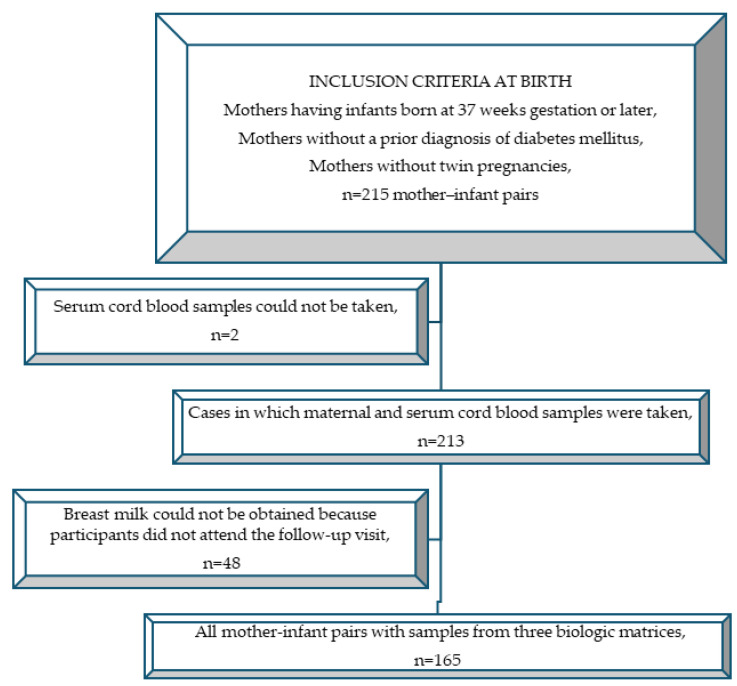
Flowchart of case selection.

A total of 215 mothers were included in this study because 25% of the mothers might not have visited the control examination. Complete maternal serum, cord serum, and breast milk samples were obtained from 81 GDM patients and 84 control participants. A total of 165 patients were analyzed (Figure 1). In two cases, analysis could not be performed because the cord sample was insufficient.

### 2.4. Study Protocol

Visit 1—Before delivery: Upon enrollment, pregnant women were informed about this study and signed consent forms prior to delivery and upon hospital admission. Sociodemographic characteristics and pregnancy-related information were collected through face-to-face interviews. Laboratory parameters obtained during pregnancy (including OGTT results, fasting blood glucose levels, and routine kidney and liver function tests) were extracted from patient files and hospital databases.

Maternal blood samples for OPN levels were collected in gel-free tubes, with 7.5 mL taken just before delivery, left at room temperature for one hour, and then centrifuged at 3000 rpm for 10 min to obtain serum, which was aliquoted into three separate Eppendorf tubes and stored at −20 °C.

Visit 2—At delivery: At the time of delivery, 5 mL cord blood samples for OPN and ANGPTL8 levels were collected in gel-free tubes. The samples were left at room temperature for one hour and then centrifuged at 3000 rpm for 10 min to obtain serum, which was aliquoted into two separate Eppendorf tubes and stored at −20 °C.

Visit 3—Postpartum days 1–2: Breastfeeding counseling was given to the mothers after delivery. Maternal skinfold thicknesses were measured with a HOLTAIN skinfold caliper (Holtain, Crosswell, UK). The mother’s weight, body water percentage, body fat percentage, muscle mass, visceral fat mass, bone mass, metabolic age, and basal metabolic rate (BMR) were measured using a TANITA^®^ BC 730 scale (TANITA, Tokyo, Japan) between 24 and 48 h after birth. The newborn’s birth weight, head circumference, and feeding method were recorded.

Visit 4—Postpartum days 10–15: The 4th visit occurred during the newborn outpatient clinic check-up on days 10–15 after delivery. The infant’s characteristics, body weight at the control examination, nutritional status, and any problems, indirect hyperbilirubinemia history, and possible GDM complications were evaluated. The mothers’ body weight and body fat percentage were measured with a TANITA^®^ BC 730 scale (TANITA, Tokyo, Japan). To measure OPN levels, while the infant was suckling from one breast, milk was obtained from the other breast using the manual milking method. Seven milliliters of breast milk was taken homogeneously into polypropylene Falcon tubes and stored at +4 °C. The sample was delivered to the laboratory for analysis within 24 h while maintaining the cold chain.

### 2.5. Storage and Analysis of Samples

Serum samples were stored at Ankara Bilkent City Hospital at −20 °C in tubes in a separate cabinet until they reached the laboratory. The samples were delivered to the relevant laboratory by maintaining the cold chain. Breast milk samples were stored in 7 mL polypropylene tubes at −80 °C. The specimens were thawed at an ambient temperature following the manufacturer’s guidelines and prepared for analysis. OPN and ANGPTL8 levels were analyzed at Diagen Biotechnological Systems Health Services and Automation Industry and Trade Joint Stock Company (Ankara, Turkey). OPN concentrations were determined using an Elabscience^®^ 96T Human OPN Enzyme Linked Immunosorbent Assay (ELISA) Kit (Elabscience, TX, USA) (Lot number: CV264T425582). ANGPTL8 concentrations were measured with an Elabscience^®^ 96T Human ANGPTL8 (Angiopoietin Like Protein 8) Enzyme Linked Immunosorbent Assay (ELISA) Kit (Elabscience, TX, USA) (Lot number: CV24BN6L7456). Measurements were made without diluting the maternal serum by diluting the cord serum 20 times for OPN, 5 times for ANGPTL8, and 216,000 times for the milk. OPN and ANGPTL8 concentrations were determined by spectrophotometric measurements.

### 2.6. Statistical Analysis

The data were coded in the IBM-SPSS 22.0 (IBM-SPSS Inc., Chicago, IL, USA) database. The acquired data underwent analysis using descriptive statistical techniques such as arithmetic mean, standard deviation, median, and percentage distributions. The means were compared between groups, and the normality of the distribution of the data was evaluated with the Shapiro–Wilk test. The “IDF” (inverse distribution function) correction, a two-step approach, has been applied to transform continuous variables that do not follow a normal distribution into a normal distribution [39]. To compare the means of two independent groups, the appropriate statistical test was chosen based on the parametric conditions. Specifically, the independent group t-test was employed when parametric conditions were satisfied, whereas the Mann–Whitney U test was utilized when parametric conditions were not met. Additionally, the chi-square test was applied to compare the percentage distributions of categorical data across different groups. Correlations between OPN levels and maternal and infant factors were evaluated with the Spearman correlation in both groups.

Maternal serum and breast milk OPN levels were divided into quartiles, with the lowest quartile being “1” and the highest quartile being “4” (Q1–Q4). The chi-square test was used to compare the percentage distributions of GDM among quartile groups. Differences in subgroups were tested with adjusted residuals. Multiple logistic regression analysis revealed an association between GDM and the OPN quartile (Q2 vs. Q1; Q3 vs. Q1; Q4 vs. Q1) after adjusting for maternal parameters with *p* < 0.1 in univariate analysis [after selecting one of the correlated variables: maternal age, prepregnancy BMI (kg/m^2^), weight gain during pregnancy (adequate vs. insufficient, excessive vs. insufficient), gestational duration (weeks), body fat percentage, bone mass (kg), and body liquid percentage]. We computed odds ratios (ORs) along with their corresponding 95% confidence intervals (CIs). *p* < 0.05 was considered to indicate statistical significance.

## 3. Results

A total of 165 patients with three complete samples—84 controls and 81 GDM patients—were included in this study. The mean age was 29.0 ± 5.6 years in the control group and 31.4 ± 5.4 years in the GDM group, and the mean age was significantly greater in the GDM group (*p* = 0.006, Table 1). While 84.5% of the participants in the control group were housewives, 77.8% of the participants in the GDM group were housewives. There was no significant difference between the two groups in terms of smoking history, spouse’s smoking history or passive smoking (Table 1).

During pregnancy, while the frequency of hypertension was greater in the GDM group, the frequencies of anemia and hypothyroidism were similar in both groups (*p* = 0.001, 0.209, and 0.183, respectively; Table 1). The GDM diagnosis was made based on the results of the OGTT performed between weeks 24 and 28 of pregnancy (Supplementary Table S1). OGTTs were not performed in 29.8% of the control group, but blood glucose was monitored. OGTTs were performed for 87.7% of mothers diagnosed with GDM, and the remainder were diagnosed with high blood glucose. The frequency of polyhydramnios was greater in the GDM group than in the control group (*p* = 0.002, Table 1). The frequency of a maternal history of GDM and T2DM in first-degree relatives was significantly greater in the GDM group (*p* = 0.007 and 0.001, respectively). Obesity history during childhood, prepregnancy, and postnatal and 10–15-day postbirth BMI measurements were significantly greater in the GDM group (*p* = 0.048, *p* < 0.0001, *p* < 0.0001, and *p* < 0.0001, respectively; Table 1).

The mean gestational duration was significantly lower in the GDM group than in the control group (37.7 ± 1.3 vs. 38.6 ± 1.2 weeks, *p* < 0.0001; Table 2). There were no significant differences in the 1st or 5th minute APGAR score, birth weight, head circumference, or chest circumference between the groups (*p* > 0.05).

Both groups of newborns had similar discharge weights on day 2 (Table 3). The postnatal fat percentage, visceral fat, and metabolic age of mothers were significantly greater in the GDM group (*p* < 0.0001, <0.0001, and <0.0001, respectively). The body liquid percentage was greater in the control group (*p* < 0.0001). Bone mass, muscle mass, and the BMR were similar between the two groups (*p* > 0.05, Table 3). In terms of skinfold thickness, measurements taken from the biceps, triceps, and subscapular regions were significantly higher in mothers in the GDM group (*p* = 0.048, 0.002, and 0.005, respectively; Table 3). Between the 10th and 15th postnatal days, the GDM group exhibited higher levels of body fat percentage, visceral fat, muscle mass, the BMR, and metabolic age compared to the control group (*p* < 0.05, Table 3).

### 3.1. OPN and ANGPTL8

The levels of maternal serum OPN and breast milk OPN were significantly higher in the GDM group than in the control group (*p* = 0.002 and 0.002, respectively; Table 2). There was no significant difference in cord OPN or ANGPTL8 levels between the groups (*p* > 0.05, Table 2).

According to sex, no significant differences were found in the levels of either ANGPTL8 or OPN among the three biomatrices in either study group (Supplementary Table S2).

At discharge, the percentage of patients with insufficient breast milk was 21.4% in the control group and 33.3% in the GDM group. During the follow-up examination, the percentage of patients with breastfeeding problems was 50.6% in the GDM group and 35.7% in the control group. When milk OPN levels were examined according to the status of breast milk insufficiency at discharge and during follow-up in both study groups, no difference was detected (Table 4). The milk OPN value was higher in the GDM group without breast milk insufficiency in both periods compared to counterparts in control group, while no significant difference was found between the two groups having breastfeeding problems. The milk OPN value was found to be higher in the control group with breastfeeding problems than the control group without problems during the follow-up examination.

In the control group, cord OPN and breast milk OPN values were higher in infants whose control weight was below the birth weight on the 10th–15th day, while cord ANGPTL8 levels were higher in the GDM group (Table 4).

In the control group, the rate of receiving a diagnosis of jaundice requiring phototherapy was 12.0%, while, in the GDM-diagnosed group, this rate was 33.8%. Among patients without jaundice in the control group, cord ANGPTL8 levels were higher than those in the GDM group (*p* = 0.029, Table 5). When patients with mild jaundice were examined, both maternal serum OPN values and milk OPN values were higher in the GDM-diagnosed group (*p* = 0.011 and 0.007, respectively). When comparing cases without jaundice or with mild jaundice in the control group with cases requiring phototherapy, it was found that milk OPN values were higher significantly in the phototherapy group (*p* = 0.021). This association was not present in the GDM group (*p* = 0.368, Table 5).

### 3.2. The Associations of Maternal Serum and Breast Milk OPN with GDM

When all mothers were included in the analysis, mothers with serum OPN-Q4 had higher percentages of GDM than mothers in other quartile levels of OPN. The GDM percentiles of mothers with Q3 and Q4 were detected to be higher than those of the other mothers (Table 6).

After adjusting for maternal factors, maternal serum OPN levels in the fourth quartile were associated with a 3.19-fold higher likelihood of GDM compared to those in the first quartile (Table 6, Supplementary Table S3). Similarly, for milk OPN levels, the likelihood of GDM was 3.83 times higher in the fourth quartile than in the first quartile.

### 3.3. Correlation Between OPN Levels and Maternal-Infant Characteristics

In both groups, there was a strong positive correlation between maternal serum OPN and breast milk OPN, as well as between maternal serum OPN and cord serum OPN (Table 7).

In the control group, head circumference was negatively correlated with maternal serum OPN, while maternal age, metabolic age, head circumference, and chest circumference were negatively correlated with cord serum OPN. Maternal weight gain status, gestational age, head circumference, and chest circumference were negatively correlated with breast milk OPN.

In the GDM group, metabolic age was positively correlated with maternal serum OPN. Maternal BMR was positively correlated with cord OPN, while maternal weight gain status, birth weight, bone mass, and muscle mass were negatively correlated with cord serum OPN. Gestational age was not associated with OPN levels.

## 4. Discussion

Our study demonstrated that maternal serum OPN and breast milk OPN levels were associated with GDM. There are studies investigating OPN levels in both Type 1 and Type 2 diabetes [12,14,16,17]. In addition, Wang et al. documented that circulating OPN levels were elevated in individuals with NAFLD as well as in those with T2DM, regardless of the presence of NAFLD [40]. However, there is a scarcity of research exploring its association specifically with GDM. One study reported that OPN levels were associated with fasting blood OPN levels independent of GDM status and were elevated in overweight/obese individuals [41]. Two cross-sectional studies have examined the relationship between OPN and GDM, and OPN levels in blood samples taken during a certain period of pregnancy have been reported [21,22]. However, we have not yet conducted a study showing how OPN levels fluctuate throughout pregnancy and during childbirth. A 2012 study conducted by Winhofer et al. examined the serum OPN levels in pregnant women between 24 and 28 weeks of gestation, finding lower OPN levels in the GDM group compared to the control group, suggesting no relationship between insulin sensitivity and OPN [21]. Similarly, another study reported no association between OPN levels and GDM in serum samples taken at the 30th week of pregnancy [22]. However, in our study, the serum OPN levels of mothers before delivery were significantly higher in the GDM group than in the control group. The reason for this difference may be our larger sample size and sampling at a different gestational age compared to previous studies.

In our study, we found that the milk OPN value was significantly higher in the control group experiencing breastfeeding problems than in the group without problems. More studies are needed to elucidate these contributing factors. Maternal stress, dietary changes and metabolic alterations may cause this discrepancy. There are previous studies reporting various changes in breast milk composition due to stress [42].

Consistent with the literature, our study revealed a greater incidence of neonatal phototherapy due to hyperbilirubinemia in the GDM group [43,44]. This highlights the need for more frequent monitoring of these infants for the early detection of jaundice and its complications. Unconjugated bilirubin levels are determined by the balance between bilirubin production and hepatic clearance. Hyperbilirubinemia can result from increased bilirubin production, decreased clearance or both, such as in cases of hemolysis [45]. Although GDM is known to be a risk factor for hyperbilirubinemia, further studies are needed to fully understand its mechanism. Additionally, we found significantly higher milk OPN values in patients who required phototherapy in the control group than in the group without jaundice or with mild jaundice (*p* = 0.021). To our knowledge, there are no studies investigating the relationship between jaundice and breast milk OPN levels. OPN derived from breast milk is resistant to digestion and can directly enter the circulation when ingested orally [46]. Therefore, it is believed that milk-derived OPN may play fundamental roles during the neonatal period. Breast milk OPN is suggested to play a role in numerous aspects of infant development, including intestinal proliferation and maturation, neurodevelopment, brain myelination, and immune system maturation [47]. One of the popular topics in recent years is that the content of breast milk may vary depending on the infant’s characteristics [48,49]. There are limited studies in the literature examining the relationship between jaundice and OPN, mainly limited to biliary atresia, one of the causes of pathological jaundice [50,51,52]. In this context, more studies are needed to elucidate the role of milk OPN in the mechanism of jaundice.

From the perspective of OPN, a limitation of our study was that we collected maternal serum only during delivery, and the mothers who were diagnosed with GDM had undergone dietary changes and insulin therapy. OPN levels in maternal serum could not be measured at the time of GDM diagnosis. However, even in this scenario, we found that OPN levels in maternal serum significantly correlated with the risk of GDM.

In our study, we also measured ANGPTL8 levels in cord blood. One study reported the involvement of ANGPTL8 in glucose and lipid metabolism [53]. A recent meta-analysis revealed an association between ANGPTL8 levels in maternal serum and GDM [54]. However, the results from studies conducted on cord blood are inconsistent [31,33,34,35]. ANGPTL8 levels are known to be associated with BMI and are increased in macrosomic infants [37]. We designed our study with a larger number of patients than did previous studies. Our results indicated no relationship between cord ANGPTL8 levels and medically well-controlled cases of GDM. There was no significant difference between the medically well-controlled GDM group and the typically healthy pregnant group in terms of birth weight. This suggests that mothers diagnosed with GDM had well-regulated glucose levels and complied with treatment, which might explain the lack of difference in ANGPTL8 levels between the two groups. A limitation of our study was the inability to measure ANGPTL8 levels in maternal serum. Therefore, we could not thoroughly examine the relationship between ANGPTL8 levels and GDM.

To the best of our understanding, this study represents the inaugural exploration into the correlations among maternal serum, cord serum, and breast milk osteopontin (OPN) levels, as well as the concurrent interactions in cord serum ANGPTL8 levels, GDM history, and body composition. There is no published report on maternal serum OPN levels during childbirth. Contrary to previous studies [21,22,23], our data revealed an association with GDM, providing a new perspective on the levels of OPN in maternal serum and breast milk.

Considering the characteristics of GDM, excessive weight gain during pregnancy and the higher birth weight of the newborn compared to the control group are commonly observed in GDM patients [55]. However, in our study, no significant difference was found between these two conditions among the groups. This might be due to the appropriate control of GDM cases.

Previous studies have shown a strong correlation between the incidence of hypertension and polyhydramnios during pregnancy and GDM [56,57,58]. Our findings in this context are consistent with the literature. The greater incidence of childhood obesity, increased skinfold thickness, BMI, body fat percentage, and visceral fat in the GDM group than in the control group supports the relationship between weight gain and GDM observed in various studies [59,60,61]. GDM is a common multifactorial metabolic condition with long-term negative effects when complications develop. Despite numerous studies, treatment is limited to diet and medication therapy given after the onset of the disease [62,63]. Therefore, to reduce the risk of GDM, excessive weight gain should be prevented in pregnant women. However, in the GDM-diagnosed group, the higher rates of pathological weight loss at newborn discharge and the higher rates of formula feeding in the control group suggest the need for more careful breastfeeding support for the GDM group. Our study indicated that better counseling services should be provided to this group.

Strengths and limitation of this Study:

This study provides novel insights into the associations between maternal serum and breast milk OPN levels and GDM. Additionally, our larger sample size and inclusion of both maternal and cord blood samples strengthen the validity and generalizability of the findings. This study also sheds light on the potential role of breast milk-derived OPN in neonatal health, particularly its possible influence on neonatal jaundice and other developmental processes.

Despite its strengths, this study has several limitations. First, the measurement of maternal serum OPN was only taken during delivery, and the potential effects of dietary changes and insulin therapy on OPN levels during the course of GDM management were not fully accounted for. The absence of OPN measurements at the time of GDM diagnosis limits our understanding of how early OPN changes might predict GDM outcomes. Additionally, while our study examined ANGPTL8 levels in cord blood, we could not assess ANGPTL8 in maternal serum, which restricts a comprehensive investigation into its role in GDM. The lack of longitudinal monitoring of OPN and ANGPTL8 levels throughout pregnancy also limits the ability to establish clear causal relationships. Finally, despite a large sample size, this study’s observational design does not allow for definitive conclusions regarding the underlying mechanisms of GDM and its impact on neonatal outcomes, necessitating further research to confirm these findings.

## 5. Conclusions

Our findings indicated that maternal serum OPN levels at delivery were higher in the GDM group compared to the non-GDM group. This difference may reflect metabolic or inflammatory changes associated with GDM or its management during pregnancy. Factors such as dietary interventions, insulin therapy, or other aspects of GDM treatment could have influenced OPN levels. However, as OPN levels were not measured at the time of GDM diagnosis, it is not possible to determine whether the observed differences were due to GDM itself or its treatment. Future studies with prospective designs are needed to determine the temporal relationship between OPN levels and GDM, as well as to explore its potential as a biomarker for monitoring metabolic changes in pregnancy.

## Figures and Tables

**Table 1:** Maternal characteristics and health risks in the study groups.

	Control (*n* = 84)	GDM (*n* = 81)	*p*
Maternal age (years)	29.0 ± 5.6	31.4 ± 5.4	0.006
Education			0.354
Middle school and below	42.9	35.8	
High school and above	57.1	64.2	
Working status	15.5	22.2	0.267
Monthly income			0.393
Below minimum wage	13.1	12.3	
Minimum wage–2× minimum wage	48.8	37.0	
2× minimum wage–3× minimum wage	32.1	40.7	
3× minimum wage and above	6.0	9.9	
Smoking rate	21.4	20.0	0.822
Husband’s smoking rate	46.4	51.9	0.486
Passive smoking	32.1	42.0	0.191
Prepregnancy BMI (kg/m^2^)	25.6 ± 5.2	30.0 ± 5.9	<0.0001
BMI, prepregnancy			<0.0001
Underweight	4.8	0.0	
Normal	48.8	25.9	
Overweight	32.1	25.9	
Obese	14.3	48.1	
Weight gain during pregnancy			0.601
Insufficient	25.0	19.8	
Adequate	25.0	30.9	
Excessive	50.0	49.4	
BMI at the 2nd day of delivery (kg/m^2^)	29.1 ± 5.6	32.2 ± 5.7	<0.0001
BMI at 10–15 days postpartum (kg/m^2^)	27.8 ± 5.4	31.1 ± 5.6	<0.0001
Oligohydramnios history in this pregnancy	1.2	6.2	0.087
Polyhydramnios history in this pregnancy	4.8	21.0	0.002
Other maternal health risks			
Childhood obesity	1.2	7.4	0.048
Hypertension	2.4	17.3	0.001
Hypothyroidism	9.5	16.0	0.209
Anemia	53.6	43.2	0.183
Mother’s parental history of Type 2 DM	38.1	59.3	0.007
Mother’s maternal history of GDM	2.4	17.3	0.001
Parental history of hypertension	36.9	58.0	0.007

The data are presented as the mean ± SD or %. Gestational diabetes mellitus: GDM; Body mass index: BMI.

**Table 2:** Infant characteristics and betatrophin and osteopontin levels in biomatrices from the study groups.

	Control (*n* = 84)	GDM (*n* = 81)	*p*
Infant characteristics			
Birth order, the first	21.4	22.2	0.902
Gestational duration, week	38.6 ± 1.2	37.7 ± 1.3	<0.0001
Sex, male	46.4	51.9	0.486
Delivery type			0.283
NSVD	8.3	6.2	
NSVD (Induction)	8.3	13.6	
C/S (Spinal Anesthesia)	81.0	72.8	
C/S (General Anesthesia)	2.4	7.4	
APGAR-1 min	7.62 ± 0.51	7.47 ± 0.67	0.108
APGAR-5 min	8.99 ± 0.45	8.80 ± 0.60	0.027
Birth weight (g)	3264 ± 426	3288 ± 555	0.758
Birth head circumference (cm)	34.9 ± 1.2	35.0 ± 1.5	0.695
Birth chest circumference (cm)	32.9 ± 1.8	32.8 ± 2.3	0.767
Cord ANGPTL8 (ng/mL)	3.33 ± 1.40	3.16 ± 1.21 *	0.405
Cord OPN (ng/mL)	219.9 ± 90.5	228.3 ± 103.6 *	0.585
Maternal serum OPN (ng/mL)	9.80 ± 3.81	11.69 ± 3.87	0.002
Breast milk OPN (mg/L)	304.9 ± 146.5	372.0 ± 129.4	0.002

* Two missing data: insufficient cord blood. The data are presented as the mean ± SD or %. Gestational diabetes mellitus: GDM; normal spontaneous vaginal delivery: NSVD; cesarean section: C/S; Appearance-Pulse-Grimace-Activity-Respiration: APGAR; betatrophin: ANGPTL8; osteopontin: OPN.

**Table 3:** Postpartum follow-up anthropometric measurements of mother–infant pairs according to study group.

	At the 2nd Day After Delivery			Between 10 and 15 Days		
	Control (*n* = 84)	GDM (*n* = 81)	*p*	Control (*n* = 84)	GDM (*n* = 81)	*p*
Newborn’s weight	3103 ± 418	3100 ± 512	0.970	3413 ± 455	3404 ± 520 ^&^	0.910
Mother’s weight (kg)	75.5 ± 15.7	84.2 ± 15.1	<0.0001	72.3 ± 15.6	81.2 ± 14.8	<0.0001
Body fat percentage	32.1 ± 7.8	37.8 ± 6.4	<0.0001	32.8 ± 7.9	37.8 ± 6.1	<0.0001
Bone mass (kg)	2.55 ± 0.32	2.61 ± 0.29	0.192	2.44 ± 0.29	3.05 ± 4.75	0.247
Body water percentage	48.3 ± 4.8	44.6 ± 4.1	<0.0001	46.3 ± 4.4	43.9 ± 3.8	<0.0001
Muscle mass (kg)	47.7 ± 6.3	48.9 ± 5.5	0.203	45.3 ± 5.9	47.2 ± 5.5	0.042
Basal metabolic rate	1539 ± 202	1592 ± 188	0.081	1472 ± 186	1538 ± 182	0.025
Metabolic age	33.9 ± 12.0	42.3 ± 8.8	<0.0001	34.7 ± 11.9	42.5 ± 8.8	<0.0001
Visceral fat accumulation (kg)	4.89 ± 2.94	7.00 ± 3.01	<0.0001	4.76 ± 2.84	6.75 ± 2.81	<0.0001
Biceps skinfold thickness (mm)	18.7 ± 8.1	21.4 ± 9.3	0.048	17.8 ± 7.7	20.0 ± 8.7	0.096
Triceps skinfold thickness (mm)	25.9 ± 10.0	30.5 ± 9.1	0.002	24.9 ± 9.5	29.2 ± 8.8	0.003
Subscapular skinfold thickness (mm)	28.8 ± 9.4	32.8 ± 8.7	0.005	27.8 ± 9.0	31.5 ± 8.2	0.007
Arm circumference (cm)	28.7 ± 4.4	30.5 ± 4.0	0.006	27.6 ± 4.2	29.7 ± 4.0	0.001
Body mass index (kg/m^2^)	29.1 ± 5.6	32.2 ± 5.7	<0.0001	27.8 ± 5.4	31.1 ± 5.6	<0.0001

^&^ Two missing data: one newborn was not brought to the follow-up examination by his mother, and one newborn could not be seen because he was diagnosed with cleft palate at another hospital. The data are presented as the mean ± SD. Gestational diabetes mellitus: GDM.

**Table 4:** Levels of breast milk OPN levels according to breastfeeding status and study groups.

		Control		GDM	
	*n*	Mean ± SD	*n*	Mean ± SD	*p*
Breast milk insufficiency at discharge					
No	66	303.7 ± 143.2	54	384.7 ± 139.9	0.002
Yes	18	309.1 ± 162.3	27	346.8 ± 102.9	0.345
	*p*	0.892		0.216	
Breast milk insufficiency at follow-up examination					
No	59	291.2 ± 145.5	51	381.8 ± 126.8	0.002
Yes	24	338.0 ± 149.6	30	355.5 ± 134.1	0.487
	*p*	0.191		0.381	
Breastfeeding problem at follow-up examination					
No	53	276.6 ± 138.9	40	366.7 ± 136.3	0.002
Yes	30	354.4 ± 151.1	41	377.2 ± 123.7	0.487
	*p*	0.020		0.716	
Infant weight: PP15-PP0					
PP15 ≥ PP0	69	283.9 ± 139.7	64	373.3 ± 134.2	<0.001
PP15 < PP0	14	407.1 ± 146.1	15 *	384.2 ± 108.2	0.634
	*p*	0.004		0.770	

* Two missing data: one newborn was not brought to the follow-up examination by his mother, and one newborn could not be seen because he was diagnosed with cleft palate at another hospital. Osteopontin: OPN; Gestational diabetes mellitus: GDM; postpartum: PP.

**Table 5:** Levels of OPN and ANGPTL8 according to presence of jaundice and study groups.

			Control		GDM *	
	Jaundice	*n*	Mean ± SD	*n*	Mean ± SD	*p*
Cord ANGPTL8	Absence	47	3.45 ± 1.32	30	2.78 ± 1.25	0.029
(ng/mL)	Mild	26	3.49 ± 1.42	22	3.45 ± 1.24	0.919
	Moderate	10	2.52 ± 1.46	26	3.38 ± 1.06	0.058
	*p*		0.126		0.076	
Cord OPN (ng/mL)	Absence	47	212.5 ± 87.0	30	229.2 ± 106.0	0.453
	Mild	26	218.8 ± 76.5	22	242.7 ± 100.4	0.354
	Moderate	10	253.3 ± 137.4	26	211.6 ± 106.0	0.337
	*p*		0.442		0.586	
Maternal serum OPN	Absence	47	9.73 ± 3.29	31	11.36 ± 4.59	0.095
(ng/mL)	Mild	26	9.25 ± 4.30	22	12.28 ± 3.47	0.011
	Moderate	10	11.40 ± 4.83	27	11.46 ± 3.34	0.967
	*p*		0.322		0.669	
Breast milk OPN	Absence	47	286.4 ± 122.5 ^a^	31	348.2 ± 111.3	0.027
(mg/L)	Mild	26	291.6 ± 147.8 ^a^	22	398.6 ± 106.9	0.007
	Moderate	10	425.0 ± 205.3 ^b^	27	378.6 ± 163.2	0.478
	*p*		0.021		0.368	

* Two missing data due to insufficient cord blood and two missing data due to absence of the follow-up examination of infant. Superscripts (a, b) indicate statistically significant differences among subgroups within variables comprising more than two subgroups (*p* < 0.05). Gestational diabetes mellitus: GDM; betatrophin: ANGPTL8; osteopontin: OPN.

**Table 6:** Association of GDM risk with quartiles of maternal serum and milk OPN levels after adjusting mother characteristics.

	*n*	GDM, %	*p*	AOR	95%CI
				Model 1 *	
Serum OPN, ng/mL			0.047		
Q1 (<7.91)	23	39.0 ^a^		1.00	
Q2 (7.91–10.99)	21	35.0 ^a^		0.84	0.29–2.38
Q3 (11.00–13.61)	22	46.8 ^a^		1.77	0.63–4.96
Q4 (>13.61)	29	60.4 ^b^		3.19	1.10–9.25
				Model 2 *	
Milk OPN, mg/L			0.001		
Q1 (<239.2)	12	29.3 ^a^		1.00	
Q2 (239.2–336.1)	15	36.6 ^a^		1.22	0.42–3.53
Q3 (336.2–433.5)	27	64.3 ^b^		4.17	1.37–12.71
Q4 (>433.5)	27	65.9 ^b^		3.83	1.27–11.61

* Multiple logistic regression analysis; and independent variables: age (years), prepregnancy BMI (kg/m^2^), weight gain during pregnancy (insufficient, adequate, excessive), gestational age (weeks), maternal body fat percentage (%), maternal body bone mass (kg), and maternal body water percentage (%). Superscripts (a, b) indicate statistically significant differences among subgroups within variables comprising more than two subgroups (*p* < 0.05). Gestational diabetes mellitus: GDM; AOR: Adjusted Odds Ratio, CI: Confidence Interval; osteopontin: OPN; quartile: Q.

**Table 7:** Correlations between maternal serum, cord blood, and milk OPN Levels and maternal-infant characteristics *.

	Mother OPN		Cord OPN		Milk OPN	
	Control	GDM	Control	GDM	Control	GDM
Maternal serum OPN (ng/mL)			0.29 (0.009)	0.45 (<0.0001)	0.35 (0.001)	0.39 (0.000)
Cord OPN (ng/mL)					0.21 (0.060)	0.43 (<0.0001)
Cord ANGPTL8 (ng/mL)	−0.06 (0.571)	−0.02 (0.871)	0.21 (0.055)	0.16 (0.172)	−0.18 (0.104)	0.22 (0.053)
Age (years)	0.07 (0.554)	0.23 (0.036)	−0.26 (0.019)	0.09 (0.421)	0.03 (0.774)	0.01 (0.967)
Gestational age (weeks)	0.01 (0.965)	0.02 (0.845)	−0.09 (0.443)	−0.09 (0.436)	−0.30 (0.005)	0.05 (0.630)
Birth weight (g)	−0.15 (0.177)	−0.18 (0.115)	−0.16 (0.139)	−0.34 (0.002)	−0.18 (0.103)	0.00 (0.973)
Head circumference (cm)	−0.25 (0.020)	−0.02 (0.838)	−0.38 (0.000)	−0.16 (0.172)	−0.22 (0.047)	−0.06 (0.601)
Chest circumference (cm)	−0.09 (0.420)	−0.10 (0.364)	−0.23 (0.039)	−0.22 (0.054)	−0.16 (0.147)	0.08 (0.483)
Newborn’s discharge weight (g)	−0.17 (0.124)	−0.21 (0.061)	−0.20 (0.074)	−0.35 (0.001)	−0.21 (0.052)	−0.02 (0.843)
Body fat percentage	−0.03 (0.800)	0.00 (0.981)	−0.19 (0.080)	−0.07 (0.525)	−0.19 (0.092)	0.14 (0.213)
Bone mass	−0.06 (0.570)	0.02 (0.859)	−0.10 (0.392)	−0.23 (0.044)	−0.15 (0.168)	−0.02 (0.844)
Body liquid percentage	0.03 (0.775)	−0.03 (0.825)	0.19 (0.091)	0.04 (0.702)	0.19 (0.077)	−0.14 (0.200)
Muscle mass	−0.07 (0.547)	0.00 (0.977)	−0.10 (0.370)	−0.25 (0.024)	−0.14 (0.202)	−0.04 (0.712)
Basal metabolic rate	−0.05 (0.660)	−0.03 (0.813)	−0.07 (0.555)	−0.25 (0.024)	−0.16 (0.140)	−0.00 (0.969)
Metabolic age (years)	−0.02 (0.869)	0.25 (0.028)	−0.27 (0.015)	−0.02 (0.877)	−0.13 (0.247)	0.12 (0.273)
Visceral fat accumulation	0.01 (0.950)	0.03 (0.817)	−0.16 (0.152)	−0.12 (0.298)	−0.15 (0.166)	0.14 (0.210)
Biceps skinfold thickness (mm)	0.02 (0.834)	0.17 (0.135)	−0.10 (0.372)	−0.07 (0.543)	−0.20 (0.066)	0.18 (0.107)
Triceps skinfold thickness (mm)	0.05 (0.630)	0.15 (0.185)	−0.14 (0.203)	−0.03 (0.798)	−0.21 (0.055)	0.17 (0.136)
Subscapular skinfold thickness (mm)	−0.06 (0.594)	0.13 (0.241)	−0.17 (0.126)	−0.03 (0.766)	−0.21 (0.055)	0.15 (0.169)
Mother weight at control (kg)	−0.19 (0.080)	−0.12 (0.285)	−0.24 (0.032)	−0.29 (0.010)	−0.26 (0.018)	−0.00 (0.975)
Body fat percentage at control	−0.02 (0.876)	−0.01 (0.914)	−0.18 (0.097)	−0.11 (0.356)	−0.21 (0.064)	0.09 (0.443)
Bone mass at control	−0.10 (0.372)	−0.06 (0.618)	−0.14 (0.209)	−0.20 (0.085)	−0.17 (0.136)	−0.21 (0.058)
Body liquid percentage at control	−0.00 (0.991)	0.01 (0.951)	0.21 (0.055)	0.10 (0.384)	0.18 (0.103)	−0.09 (0.419)
Muscle mass at control	−0.04 (0.710)	−0.03 (0.789)	−0.13 (0.235)	−0.20 (0.072)	−0.09 (0.406)	−0.02 (0.859)
Basal metabolic rate at control	−0.09 (0.443)	−0.05 (0.649)	−0.09 (0.403)	−0.18 (0.120)	−0.18 (0.096)	0.02 (0.854)
Metabolic age at control	−0.01 (0.959)	0.19 (0.087)	−0.26 (0.020)	−0.07 (0.560)	−0.13 (0.258)	0.08 (0.505)
Visceral fat accumulation at control	0.01 (0.927)	0.03 (0.763)	−0.16 (0.160)	−0.10 (0.380)	−0.17 (0.135)	0.13 (0.240)
Biceps skinfold thickness at control (mm)	0.04 (0.747)	0.17 (0.122)	−0.08 (0.465)	−0.04 (0.728)	−0.18 (0.098)	0.20 (0.076)
Triceps skinfold thickness at control (mm)	0.06 (0.563)	0.12 (0.308)	−0.12 (0.274)	−0.03 (0.803)	−0.19 (0.083)	0.13 (0.264)
Subscapular skinfold thickness at control (mm)	−0.05 (0.652)	0.15 (0.198)	−0.16 (0.136)	−0.01 (0.931)	−0.21 (0.054)	0.16 (0.164)
Prepregnancy BMI (kg/m^2^)	−0.12 (0.282)	0.00 (0.973)	−0.06 (0.610)	−0.11 (0.329)	−0.06 (0.565)	0.08 (0.466)
Postpregnancy BMI (kg/m^2^)	0.00 (0.974)	−0.01 (0.937)	−0.07 (0.553)	−0.17 (0.132)	−0.14 (0.199)	0.10 (0.372)
BMI at control (kg/m^2^)	0.01 (0.906)	−0.02 (0.879)	−0.06 (0.597)	−0.15 (0.200)	−0.12 (0.263)	0.11 (0.346)
Weight change during pregnancy (kg)	−0.08 (0.488)	0.09 (0.406)	−0.09 (0.442)	−0.18 (0.106)	−0.26 (0.018)	0.17 (0.139)

* r(*p*) Gestational diabetes mellitus: GDM; osteopontin: OPN; betatrophin: ANGPTL8; BMI: Body mass index.

## Data Availability

The raw data supporting the conclusions of this article will be made available by the corresponding authors upon request.

## Supplementary Materials

The following supporting information can be downloaded at https://www.mdpi.com/article/10.3390/nu16244334/s1, Supplementary File: Osteopontin Levels in Maternal Serum, Cord Blood, and Breast Milk According to Gestational Diabetes Mellitus: A Case-Control Study, Survey Record Form; Table S1: Gestational diabetes history in the study groups; Table S2: Levels of OPN and ANGPTL8 according to infant sex and study groups; Table S3: Association of GDM risk with maternal characteristics and OPN levels (Model 1: maternal serum, model 2: breast milk) *.

## References

[1] Alberti K.G., Zimmet P.Z. (1998). Definition, diagnosis and classification of diabetes mellitus and its complications. Part 1: Diagnosis and classification of diabetes mellitus provisional report of a WHO consultation. Diabet. Med..

[2] Catalano P.M. (2014). Trying to understand gestational diabetes. Diabet. Med..

[3] Feig D.S., Hwee J., Shah B.R., Booth G.L., Bierman A.S., Lipscombe L.L. (2014). Trends in incidence of diabetes in pregnancy and serious perinatal outcomes: A large, population-based study in Ontario, Canada, 1996–2010. Diabetes Care.

[4] Dugas C., Perron J., Kearney M., Mercier R., Tchernof A., Marc I., Weisnagel S.J., Robitaille J. (2017). Postnatal Prevention of Childhood Obesity in Offspring Prenatally Exposed to Gestational Diabetes mellitus: Where Are We Now?. Obes. Facts.

[5] International Diabetes Federation (2021). IDF Diabetes Atlas.

[6] Lewandowska M. (2021). Gestational diabetes mellitus (GDM) risk for declared family history of diabetes, in combination with BMI categories. Int. J. Environ. Res. Public Health.

[7] Li Y., Ren X., He L., Li J., Zhang S., Chen W. (2020). Maternal age and the risk of gestational diabetes mellitus: A systematic review and meta-analysis of over 120 million participants. Diabetes Res. Clin. Pract..

[8] Plows J.F., Stanley J.L., Baker P.N., Reynolds C.M., Vickers M.H. (2018). The pathophysiology of gestational diabetes mellitus. Int. J. Mol. Sci..

[9] Lund S.A., Giachelli C.M., Scatena M. (2009). The role of osteopontin in inflammatory processes. J. Cell Commun. Signal..

[10] Sun L.-L., Liu T.-J., Li L., Tang W., Zou J.-J., Chen X.-F., Zheng J.-Y., Jiang B.-G., Shi Y.-Q. (2017). Transplantation of betatrophin-expressing adipose-derived mesenchymal stem cells induces β-cell proliferation in diabetic mice. Int. J. Mol. Med..

[11] Scatena M., Liaw L., Giachelli C.M. (2007). Osteopontin: A multifunctional molecule regulating chronic inflammation and vascular disease. Arter. Thromb. Vasc. Biol..

[12] Icer M.A., Gezmen-Karadag M. (2018). The multiple functions and mechanisms of osteopontin. Clin. Biochem..

[13] Sarosiek K., Jones E., Chipitsyna G., Al-Zoubi M., Kang C., Saxena S., Gandhi A.V., Sendiky J., Yeo C.J., Arafat H.A. (2015). Osteopontin (OPN) isoforms, diabetes, obesity, and cancer; what is one got to do with the other? A new role for OPN. J. Gastrointest. Surg..

[14] Gong Q., Chipitsyna G., Gray C.F., Anandanadesan R., Arafat H.A. (2009). Expression and regulation of osteopontin in type 1 diabetes. Islets.

[15] Katakam A., Chipitsyna G., Gong Q., Vancha A., Gabbeta J., Arafat H. (2005). Streptozotocin (STZ) mediates acute upregulation of serum and pancreatic osteopontin (OPN): A novel islet-protective effect of OPN through inhibition of STZ-induced nitric oxide production. J. Endocrinol..

[16] Barchetta I., Alessandri C., Bertoccini L., Cimini F., Taverniti L., Di Franco M., Fraioli A., Baroni M., Cavallo M. (2016). Increased circulating osteopontin levels in adult patients with type 1 diabetes mellitus and association with dysmetabolic profile. Eur. J. Endocrinol..

[17] Talat M.A., Sherief L.M., El-Saadany H.F., Rass A.A., Saleh R.M., Sakr M.M. (2016). The Role of Osteopontin in the Pathogenesis and Complications of Type 1 Diabetes Mellitus in Children. J. Clin. Res. Pediatr. Endocrinol..

[18] Nomiyama T., Perez-Tilve D., Ogawa D., Gizard F., Zhao Y., Heywood E.B., Jones K.L., Kawamori R., Cassis L.A., Tschöp M.H. (2007). Osteopontin mediates obesity-induced adipose tissue macrophage infiltration and insulin resistance in mice. J. Clin. Investig..

[19] Gómez-Ambrosi J., Catalán V., Ramírez B., Rodríguez A., Colina I., Silva C., Rotellar F., Mugueta C., Gil M.a.J., Cienfuegos J.A. (2007). Plasma osteopontin levels and expression in adipose tissue are increased in obesity. J. Clin. Endocrinol. Metab..

[20] Fathy M.A., Elwany N.E., Habib M.A., Mohamed N.M., Khalil S.S. (2023). Omentin-1 a Novel Approach Ameliorates Glycemic State, Inflammation and Osteopontin Level in GDM Rat Model: PI3K/AKT/GSK-3 pathway. Zagazig Univ. Med. J..

[21] Winhofer Y., Kiefer F.W., Handisurya A., Tura A., Klein K., Schneider B., Marculescu R., Wagner O.F., Pacini G., Luger A. (2012). CTX (crosslaps) rather than osteopontin is associated with disturbed glucose metabolism in gestational diabetes. PLoS ONE.

[22] Saucedo R., Rico G., Vega G., Basurto L., Cordova L., Galvan R., Hernandez M., Puello E., Zarate A. (2015). Osteocalcin, under-carboxylated osteocalcin and osteopontin are not associated with gestational diabetes mellitus but are inversely associated with leptin in non-diabetic women. J. Endocrinol. Investig..

[23] Saklamaz A., Akyildiz M., Kasap E., Cengiz H. (2017). Osteopontin levels do not increase in gestational diabetes mellitus. Ege Tıp Dergisi.

[24] Aksan A., Erdal I., Yalcin S.S., Stein J., Samur G. (2021). Osteopontin Levels in Human Milk Are Related to Maternal Nutrition and Infant Health and Growth. Nutrients.

[25] Schack L., Lange A., Kelsen J., Agnholt J., Christensen B., Petersen T.E., Sørensen E.S. (2009). Considerable variation in the concentration of osteopontin in human milk, bovine milk, and infant formulas. J. Dairy Sci..

[26] Fu Z., Yao F., Abou-Samra A.B., Zhang R. (2013). Lipasin, thermoregulated in brown fat, is a novel but atypical member of the angiopoietin-like protein family. Biochem. Biophys. Res. Commun..

[27] Guo Q., Cao S., Wang X. (2022). Betatrophin and Insulin Resistance. Metabolites.

[28] Erol O., Ellidağ H.Y., Ayık H., Özel M.K., Derbent A.U., Yılmaz N. (2015). Evaluation of circulating betatrophin levels in gestational diabetes mellitus. Gynecol. Endocrinol..

[29] Ebert T., Kralisch S., Wurst U., Lössner U., Kratzsch J., Blüher M., Stumvoll M., Tönjes A., Fasshauer M. (2015). Betatrophin levels are increased in women with gestational diabetes mellitus compared to healthy pregnant controls. Eur. J. Endocrinol..

[30] Trebotic L.K., Klimek P., Thomas A., Fenzl A., Leitner K., Springer S., Kiefer F.W., Kautzky-Willer A. (2015). Circulating betatrophin is strongly increased in pregnancy and gestational diabetes mellitus. PLoS ONE.

[31] Wawrusiewicz-Kurylonek N., Telejko B., Kuzmicki M., Sobota A., Lipinska D., Pliszka J., Raczkowska B., Kuc P., Urban R., Szamatowicz J. (2015). Increased maternal and cord blood betatrophin in gestational diabetes. PLoS ONE.

[32] Huang Y., Fang C., Ma Z., Guo H., Wang R., Hu J. (2016). Betatrophin Levels were Increased in Pregnant Women with or without Gestational Diabetes Mellitus and Associated with Beta Cell Function. Rev. Bras. Ginecol. Obstet..

[33] Xie X., Gao H., Wu S., Zhao Y., Du C., Yuan G., Ning Q., McCormick K., Luo X. (2016). Increased Cord Blood Betatrophin Levels in the Offspring of Mothers with Gestational Diabetes. PLoS ONE.

[34] Martinez-Perez B., Ejarque M., Gutierrez C., Nuñez-Roa C., Roche K., Vila-Bedmar R., Ballesteros M., Redondo-Angulo I., Planavila A., Villarroya F. (2016). Angiopoietin-like protein 8 (ANGPTL8) in pregnancy: A brown adipose tissue-derived endocrine factor with a potential role in fetal growth. Transl. Res..

[35] Yang F., Yang W., Wang G., Liu Y., Jin J. (2021). Association of betatrophin amounts with 25-(OH)D levels in patients with gestational diabetes mellitus. Medicine.

[36] Pan R., Zhang H., Yu S., Deng J., Ma S., Li Y., Yuan G., Wang J. (2019). Betatrophin for diagnosis and prognosis of mothers with gestational diabetes mellitus. J. Int. Med. Res..

[37] Zheng J., Liu J., Hong B.S., Ke W., Huang M., Li Y. (2020). Circulating betatrophin/ANGPTL8 levels correlate with body fat distribution in individuals with normal glucose tolerance but not those with glucose disorders. BMC Endocr. Disord..

[38] Zheng J., Pan Y., Ke R., Chen H., Hu X., Wang D., Huang Y. (2020). The relationship between serum betatrophin and early growth of fetal macrosomia. Chin. J. Neonatol..

[39] Templeton G.F. (2011). A two-step approach for transforming continuous variables to normal: Implications and recommendations for IS research. Commun. Assoc. Inf. Syst..

[40] Wang C., He M., Peng J., Li S., Long M., Chen W., Liu D., Yang G., Zhang L. (2020). Increased plasma osteopontin levels are associated with nonalcoholic fatty liver disease in patients with type 2 diabetes mellitus. Cytokine.

[41] Ahmad R., Al-Mass A., Al-Ghawas D., Shareif N., Zghoul N., Melhem M., Hasan A., Al-Ghimlas F., Dermime S., Behbehani K. (2013). Interaction of osteopontin with IL-18 in obese individuals: Implications for insulin resistance. PLoS ONE.

[42] Hart S., Boylan L.M., Border B., Carroll S.R., McGunegle D., Lampe R.M. (2004). Breast milk levels of cortisol and Secretory Immunoglobulin A (SIgA) differ with maternal mood and infant neuro-behavioral functioning. Infant Behav. Dev..

[43] Zanardo V., Suppiej A., Tortora D., Sandri A., Severino L., Mezzalira L., Grego L., Straface G. (2023). Trajectory of serum bilirubin in offspring of women with gestational diabetes mellitus. Diabetes Res. Clin. Pract..

[44] Soumya P. (2022). A Study of Maternal Risk Factors Associated with Development of Neonatal Jaundice: A Cross Sectional Study.

[45] Wolkoff A.W., Berk P.D. (2017). Bilirubin metabolism and jaundice. Schiff’s Dis. Liver.

[46] Liu L., Jiang R., Liu J., Lönnerdal B. (2020). The bovine Lactoferrin-Osteopontin complex increases proliferation of human intestinal epithelial cells by activating the PI3K/Akt signaling pathway. Food Chem..

[47] Jiang R., Lönnerdal B. (2020). Effects of milk osteopontin on intestine, neurodevelopment, and immunity. Milk Mucosal Immun. Microbiome Impact Neonate.

[48] Gidrewicz D.A., Fenton T.R. (2014). A systematic review and meta-analysis of the nutrient content of preterm and term breast milk. BMC Pediatr..

[49] Bilgiç F.Ş., Bozkurt G., Çoban A. (2023). The Relationship Between the Characteristics of the Newborn and the Nutrient Content of Breast Milk. Fenerbahçe Üni. Sağlık Bilim. Derg..

[50] Honsawek S., Vejchapipat P., Chongsrisawat V., Thawornsuk N., Poovorawan Y. (2011). Association of circulating osteopontin levels with clinical outcomes in postoperative biliary atresia. Pediatr. Surg. Int..

[51] Honsawek S., Chayanupatkul M., Chongsrisawat V., Vejchapipat P., Poovorawan Y. (2010). Increased osteopontin and liver stiffness measurement by transient elastography in biliary atresia. World J. Gastroenterol..

[52] Aldeiri B., Si T., Huang Z., Torner N., Ma Y., Davenport M., Hadzic N. (2023). Matrix Metalloproteinase-7 and Osteopontin Serum Levels as Biomarkers for Biliary Atresia. J. Pediatr. Gastroenterol. Nutr..

[53] Zhang R., Abou-Samra A.B. (2014). A dual role of lipasin (betatrophin) in lipid metabolism and glucose homeostasis: Consensus and controversy. Cardiovasc. Diabetol..

[54] Pérez-López F.R., Yuan J., Sánchez-Prieto M., López-Baena M.T., Pérez-Roncero G.R., Varikasuvu S.R. (2023). Maternal and cord blood betatrophin (angiopoietin-like protein 8) in pregnant women with gestational diabetes and normoglycemic controls: A systematic review, meta-analysis, and meta-regression. Diabetes/Metab. Res. Rev..

[55] Brunner S., Stecher L., Ziebarth S., Nehring I., Rifas-Shiman S.L., Sommer C., Hauner H., von Kries R. (2015). Excessive gestational weight gain prior to glucose screening and the risk of gestational diabetes: A meta-analysis. Diabetologia.

[56] Rajashree D., Paunikar V.M. (2019). Maternal complications of gestational diabetes mellitus. Natl. J. Physiol. Pharm. Pharmacol..

[57] Carpenter M.W. (2007). Gestational diabetes, pregnancy hypertension, and late vascular disease. Diabetes Care.

[58] Çetin C., Demir C. (2015). Gestational Diabetes Screening During Pregnancy. Arşiv Kaynak Tarama Derg..

[59] Alwash S.M., McIntyre H.D., Najman J., Mamun A. (2022). Triceps skinfold thickness and body mass index and the risk of gestational diabetes mellitus: Evidence from a multigenerational cohort study. Obes. Res. Clin. Pract..

[60] Cremona A., O’Gorman C.S., Ismail K.I., Hayes K., Donnelly A.E., Hamilton J., Cotter A. (2021). A risk-prediction model using parameters of maternal body composition to identify gestational diabetes mellitus in early pregnancy. Clin. Nutr. ESPEN.

[61] Aydin B., Yalçin S.S. (2022). Changes in maternal anthropometric measurements in the first postpartum month and associated factors. Am. J. Hum. Biol..

[62] American Diabetes Association (2020). 14. Management of Diabetes in Pregnancy: Standards of Medical Care in Diabetes-2020. Diabetes Care.

[63] Buchanan T.A., Xiang A.H., Page K.A. (2012). Gestational diabetes mellitus: Risks and management during and after pregnancy. Nat. Rev. Endocrinol..

