# Supplementary material for: Osteopontin Levels in Maternal Serum, Cord Blood, and Breast Milk According to Gestational Diabetes Mellitus: A Case-Control Study
Source: Nutrients. 2024 Dec 16;16(24):4334. doi: 10.3390/nu16244334 (PMC11677047; doi:10.3390/nu16244334)
Supplement: Supplementary file 1 [file nutrients-16-04334-s001.zip › Supplementary File.pdf]

Supplementary File

Osteopontin Levels in Maternal Serum, Cord Blood, and Breast Milk According to Gestational Diabetes Mellitus: A Case–Control Study

Survey Record Form

Mother registration number:

VISIT-1:
Date:

Mother's date of birth:
Maternal age:

Educational background:
a. No reading or writing
b. Primary school
c. Middle school
d. High school
e. Associate degree
f. License
g. Degree
h. Phd

Job:

Working status:
a. I worked; I quit my job ….month ago
b. I am working
c. I did not work

Monthly income level
a. Minimum wage and below
b. Minimum wage-2*Minimum wage
c. 2*Minimum wage-3*Minimum wage
d. 3*Minimum wage and above

Regarding your pregnancy
BW before pregnancy:
Weight gained during pregnancy:

Time to perform the OGGT:
Which OGGT was performed?.......

According to the OGTT results:
(1) No treatment (2) Diet (3) Diet+Insulin

Did you take any vitamin/mineral/drug supplements during your pregnancy?
Yes No
If so, what are their names?
what stage of pregnancy did you take it?

how long you have been taking it
how many times a day you took it
how many you took per day

If you have had a pregnancy before, has a sugar loading test been performed?
a. 1st pregnancy Yes No
b. 2nd pregnancy Yes No
c. 3rd pregnancy Yes No

If you have been pregnant before, do you have a history of giving birth to a baby weighing over 4000 grams?
a. 1st pregnancy Yes No
b. 2nd pregnancy Yes No
c. 3rd pregnancy Yes No

Does your spouse smoke? Yes No
If so, write down how many years you have been using it.
If yes, write down how many you use per day.

Is smoking allowed in your environment?

Yes No

If smoking, please indicate how many years you have been exposed to it

Can I learn about the health problems you have experienced thus far?

	Childhood - before pregnancy	Adult period	Occurring in previous pregnancies	Occurring in this pregnancy
Do you have a history of diabetes?	**no yes**	**no yes**	**no yes**	**no yes**
Have you used any medication for sugar regulation (Glifor, etc.) Yes No	**no yes**	**no yes**	**no yes**	**no yes**
Hypertension	**no yes**	**no yes**	**no yes**	**no yes**
Heart disease	**no yes**	**no yes**	**no yes**	**no yes**
Hypothyroidism	**no yes**	**no yes**	**no yes**	**no yes**
Hyperthyroidism	**no yes**	**no yes**	**no yes**	**no yes**
Anemia	**no yes**	**no yes**	**no yes**	**no yes**
Polycystic Ovary Syndrome	**no yes**	**no yes**	**no yes**	**no yes**
Obesity	**no yes**	**no yes**	**no yes**	**no yes**
Oligohydramnios			**no yes**	**no yes**
Polyhydramnios			**no yes**	**no yes**
Have you smoked?	**no yes**	**no yes**	**no yes**	**no yes**

Do any of your close relatives have any of the following conditions?

		If so, who has it?
A history of childhood diabetes	**no yes**	
History of diabetes occurring in adulthood	**no yes**	
History of diabetes occurring during pregnancy	**no yes**	
Heart disease	**no yes**	
Hypothyroidism	**no yes**	
Hyperthyroidism	**no yes**	
Anemia	**no yes**	
Obesity	**no yes**	
Polycystic Ovary Syndrome	**no yes**	
History of other illness	……..	

2. VISIT (at delivery, collecting sample)

Kord blood

3. VISIT (PP days 1-2)

Postpartum (hr:…….)

Pregnancy history:
Number of pregnancies:
Number of miscarriages/curettages/if any:
Number of living children:
Pregnancy week:
Date of birth:
Birth time:
Baby's gender: Girl Boy
Birth weight:
Head circumference:
Chest circumference:

Birth type:
- NSVY (induction)
- C/S (spinal)
- C/S (epidural)
- C/S (general)

APGAR-1 min…….. 5 min……..

Did you receive prenatal breastfeeding training? Yes No

Where was your baby monitored after birth?
The Intensive Care Mother's Side

Pathologies in the baby's physical examination

When did you first feel fullness in your breasts?
- Prenatal
- In the first hour after birth
- 1 hour-1 day
- 1-3 days
- for more than 3 days

When was skin-to-skin contact made?
- In the first hour
- More than 1 hour…..per hour

When did you first start breastfeeding?
- In the first hour
- More than 1 hour…..per hour

Have you had breastfeeding problems?
Yes No
If yes, what are the reasons?

-Insufficient/absent breast milk/concern about insufficient milk/thinking that the baby is not full/insufficient weight gain
-The baby does not suck enough/does not want/does not sleep/difficulty in sucking
-The mother's lack of knowledge of breastfeeding techniques/lack of knowledge and experience/needs for training and assistance
-Prematurity/inability to hold the breast fully/baby small/weak/having health problems
-Nipple size/Flat/inverted
-Redness/temperature changes in the nipple. Cracked/sore/bleeding on the nipple.
-Pain/tenderness in the breasts
-Swelling/fullness/engorgement
-Mastitis
-Excessive milk release
-Clogged milk duct
-Difficulty breastfeeding after cesarean section
-Delayed feeding/breastfeeding
-Cleft palate
-Mothers' illness/medication use
-Difficulty calming the baby
Other

Mother BW: …. Height:…..

As a result of the TANİTA measurement;

-

-

Arm circumference:………….

Skinfold thickness measurement;

Biceps……….

Triceps………….

Subscapular……………

4. VISIT (outpatient clinic check in the first 10-15 days)

Case number:
Postpartum (date:…….)

Weight:
Jaundice:
Need for phototherapy: Yes No
Does your baby have a problem?

Has your baby been given formula?
Yes No
If given, how many days in total were given?
How many times a day was it given?
How much was given daily (ml)

Who suggested starting formula?
Healthcare personnel Myself

Have you given your baby liquids other than breast milk or formula?
Yes No
If you gave, what was given?

Did your baby have low blood sugar while lying down? Yes No
Was there any ICU admission for this reason?
Yes No

How old was your baby when you were discharged?
First 24-48 hours
48-72 hours
Above 72 hours

Your baby's discharge weight

How was your baby fed at the time of discharge?
Breast milk formula

How is your baby feeding now?
Breast milk formula

Have you given formula to your baby in the last 24 hours? Yes No

Have you had any problems with breastfeeding? Yes No
If yes, what are the reasons?
-Insufficient/absent breast milk/concern about insufficient milk/thinking that the baby is not full/insufficient weight gain
-The baby does not suck enough/does not want/does not sleep/difficulty in sucking
-The mother's lack of knowledge of breastfeeding techniques/lack of knowledge and experience/needs for training and assistance
-Prematurity/inability to hold the breast fully/baby small/weak/having health problems
-Nipple size/Flat/inverted
-Redness/temperature changes in the nipple. Cracked/sore/bleeding on the nipple.
-Pain/tenderness in the breasts
-Swelling/fullness/engorgement
-Mastitis
-Excessive milk release
-Clogged milk duct
-Difficulty breastfeeding after cesarean section
-Delayed feeding/breastfeeding
-Cleft palate
-Mothers' illness/medication use
-Difficulty calming the baby
Other

Physical examination findings

If present, write results;
ECHO…………..
Abdominal USG…………..
Cranial USG……………..

Mother BW: …. Height:…..
As a result of the TANİTA measurement;

-

-

Arm circumference:………….

Skinfold thickness measurement;

Biceps……….

Triceps………….

Subscapular……………
