# Supplementary material for: Osteopontin Levels in Maternal Serum, Cord Blood, and Breast Milk According to Gestational Diabetes Mellitus: A Case-Control Study
Source: Nutrients. 2024 Dec 16;16(24):4334. doi: 10.3390/nu16244334 (PMC11677047; doi:10.3390/nu16244334)
Supplement: Supplementary file 1 [file nutrients-16-04334-s001.zip › Supplementary Tables.pdf]

Supplementary Table S1. Gestational diabetes mellitus history in the study groups

	Control (n=84)	GDM (n=81)	
Oral Glucose Tolerance Test			
Not performed	29.8	0.0	
50 gr, normal	42.9	0.0	
75 gr, normal	27.4	0.0	
50 gr, GDM	0.0	14.8	
50 gr and 100 gr, GDM	0.0	21.0	
75 gr, GDM	0.0	51.9	
Not performed, elevated blood glucose	0.0	12.3	
Oral Glucose Tolerance Test in any pregnancy			0.741
First pregnancy	21.4	25.9	
Pregnancy, test not conducted	19.0	21.0	
Pregnancy, test normal	58.3	50.6	
Pregnancy, test positive	1.2	2.5	
Diabetes history in any pregnancy	1.2	100.0	<0.0001
Diabetic medication usage history in any pregnancy	2.4	65.4	<0.0001
Macrosomia in any pregnancy			0.471
First pregnancy, normal	21.4	25.9	
At least 2nd pregnancy, normal	71.4	63.0	
At least 1 macrosomia	7.1	11.1	
Oligohydramnios history in previous pregnancies	4.8	1.3	0.191
Oligohydramnios history in this pregnancy	1.2	6.2	0.087
Polyhydramnios history in previous pregnancies	7.1	3.8	0.340
Polyhydramnios history in this pregnancy	4.8	21.0	0.002

GDM: Gestational diabetes mellitus

Supplementary Table S2. Levels of OPN and ANGPTL8 according to infant sex and study groups

		Control		GDM		
		n	mean±SD	n	mean±SD	p
	**Sex**					
Cord ANGPTL8	Female	45	3.22±1.34	38	3.11±1.00	0.699
(ng/mL)	Male	39	3.45±1.47	41	3.19±1.39	0.418
		p	0.447		0.782	
Cord OPN	Female	45	221.7±95.8	38	236.1±107.4	0.521
(ng/mL)	Male	39	217.9±85.20	41	221.0±100.8	0.884
		p	0.851		0.522	
Maternal serum OPN	Female	45	9.51±3.53	39	11.58±4.34	0.018
(ng/mL)	Male	39	10.13±4.13	42	11.78±3.44	0.053
		p	0.460		0.817	
Breast milk OPN	Female	45	320.6±137.7	39	384.1±135.5	0.037
(mg/L)	Male	39	286.6±155.8	42	360.8±124.0	0.020
		p	0.292		0.422	

GDM: Gestational diabetes mellitus; SD: standard deviation; ANGPTL8: betatrophin; OPN: osteopontin

Supplementary Table S3. Association of Gestational diabetes mellitus risk with maternal characteristics and OPN levels

	OR	95% CI	p
**Model 1***			
**Age**	1.03	0.96-1.10	0.486
**Prepregnancy body mass index**	1.12	0.98-1.28	0.108
**Weight gain during pregnancy**			0.640
Adequate vs. Insufficient	1.54	0.49-4.82	0.456
Excessive vs. Insufficient	1.06	0.30-3.71	0.932
**Gestational duration**	0.59	0.43-0.82	0.002
**Maternal body fat percentage**	0.97	0.75-1.26	0.819
**Maternal body bone mass**	0.60	0.10-3.78	0.588
**Maternal body water percentage**	0.88	0.60-1.30	0.524
**Mother's serum OPN,** ng/mL			0.065
Q2 vs Q1	0.84	0.29-2.38	0.737
Q3 vs Q1	1.77	0.63-4.96	0.277
Q4 vs. Q1	3.19	1.10-9.25	0.033
**Constant**	24.6×10^9		0.094
**Model 2****			
**Age**	1.05	0.98-1.13	0.141
**Prepregnancy body mass index**	1.10	0.96-1.26	0.170
**Weight gain**			0.446
Adequate vs. Insufficient	2.13	0.66-6.87	0.205
Excessive vs. Insufficient	1.72	0.47-6.31	0.414
**Gestational duration**	0.63	0.45-0.88	0.006
**Maternal body fat percentage**	0.95	0.72-1.24	0.682
**Maternal body bone mass**	0.72	0.11-4.84	0.733
**Maternal body liquid percentage**	0.84	0.56-1.27	0.410
**Breast milk OPN,** mg/L			0.016
Q2 vs Q1	1.22	0.42-3.53	0.717
Q3 vs Q1	4.17	1.37-12.71	0.012
Q4 vs. Q1	3.83	1.27-11.61	0.017
**Constant**	$11.3 \text{ X } 10^9$		0.122

*Model 1: maternal serum, **Model 2: breast milk

OPN: osteopontin; OR: odds ratio; CI: confidence interval; Q: quartile
